# Classification and Identification of Foodborne Bacteria in Beef by Utilising Surface-Enhanced Raman Spectroscopy Coupled with Chemometric Methods

**DOI:** 10.3390/foods13223688

**Published:** 2024-11-19

**Authors:** Huixin Zuo, Yingying Sun, Mingming Huang, Stephanie Marie Fowler, Jing Liu, Yimin Zhang, Yanwei Mao

**Affiliations:** 1 College of Food Science and Engineering, Shandong Agricultural University, Tai’an 271018, China; hxzuo@sdau.edu.cn (H.Z.); yysun1022@163.com (Y.S.); hades3709@126.com (M.H.); 13853002920@163.com (J.L.); ymzhang@sdau.edu.cn (Y.Z.); 2 National R & D Centre for Beef Processing Technology, Tai’an 271018, China; 3 College of Intelligent Engineering, Taishan Science and Technology College, Tai’an 271018, China; 4 NSW Department of Primary Industries, Centre for Red Meat and Sheep Development, P.O. Box 129, Cowra, NSW 2794, Australia; steph.fowler@dpi.nsw.gov.au

**Keywords:** foodborne pathogenic bacteria, surface-enhanced Raman spectroscopy, silver nanoparticles, chemometrics, classification identification, bacterial typing

## Abstract

The detection and classification of foodborne pathogenic bacteria is crucial for food safety monitoring, consequently requiring rapid, accurate and sensitive methods. In this study, the surface-enhanced Raman spectroscopy (SERS) technique coupled with chemometrics methods was used to detect and classify six kinds of foodborne pathogenic bacteria, including *Salmonella typhimurium* (*S*. *typhimurium*), *Escherichia coli* (*E*. *coli*) O157:H7, *Staphylococcus aureus* (*S*. *aureus*), *Listeria monocytogenes* (*L*. *monocytogenes*), *Listeria innocua* (*L*. *innocua*), and *Listeria welshimeri* (*L*. *welshimeri*). First, silver nanoparticles (AgNPs) with different particle sizes were prepared as SERS-enhanced substrates by changing the concentration of sodium citrate, and the volume ratio of silver nanosol to bacterial solution was optimised to obtain the optimal SERS signal. Then, principal component analysis (PCA) and hierarchical cluster analysis (HCA) were used to classify the SERS spectra of six bacteria at three classification levels (Gram type level, genus level and species level), and appropriate classification models were established. Finally, these models were validated on 540 spectra using linear discriminant analysis (LDA), achieving an average accuracy of 95.65%. Overall, it was concluded that the SERS technique combined with chemometrics methods could achieve the rapid detection and classification identification of foodborne pathogenic bacteria, providing an effective means for food safety monitoring.

## 1. Introduction

With the development of the food industry, people require not only delicious and natural but also healthy and safe food. However, foodborne diseases have occurred frequently worldwide, posing a serious threat to human health. A major cause of foodborne diseases is the contamination of food by foodborne pathogenic bacteria and other microorganisms. These bacteria species have demonstrated severe health effects after consuming foods and high mortality related to acute cases [1]. Bacteria are widely distributed in nature, and food can be contaminated by various bacteria during production, processing, storage and transportation. Therefore, timely and effective detection of bacterial content in food is of great importance for the prevention and control of foodborne diseases. At present, there are many methods for the detection of foodborne pathogenic bacteria in food, among which the most used is the culture method combined with colony counting. Although this method is accurate and reliable, it is labour and time intensive [2], which cannot satisfy the demand for rapid qualitative and quantitative detection. In addition, there are some methods based on immunology, molecular biology or bioluminescence [3], which are sensitive and can detect one or more bacteria simultaneously but generally involve complex methodologies and consequently have a high cost. Therefore, there is an urgent need for a detection method that can achieve a rapid, sensitive, accurate and simple detection of foodborne pathogenic bacteria.

Surface-enhanced Raman spectroscopy (SERS) is an enhanced technique based on Raman spectroscopy, as the interaction between metal nanoparticles or metal surfaces with nanostructures (called SERS substrates) and target molecules results in an average intensity enhancement of seven orders of magnitude compared with Raman scattering [4,5]. Generally speaking, metals, especially silver or gold, are often used as SERS substrates in the excitation range of visible light or near-infrared light, which enables single-molecule detection possible by SERS [6]. SERS has the advantage of being insensitive to water and, therefore, has the potential to be used for the detection and analysis of foodborne microorganisms.

When using the SERS technique to study foodborne microorganisms, Fan et al. [7] showed great potential for detecting food- and water-borne pathogens. By introducing silver nanoparticles (AgNPs) into cells, they proposed an improved SERS method that rapidly detected and distinguished bacteria at the single-cell level and found that different species and strains showed distinct vibrational spectral features, which provided a sensitive and effective tool for food and water safety control [7]. Furthermore, others, such as Efrima et al. [8] and Zeiri et al. [9], used SERS to classify bacteria and identified several spectral regions that were important for bacterial identification, while Zhao et al. [10] studied the spectra of *Escherichia coli* (*E*. *coli*) and *Salmonella typhimurium* (*S*. *typhimurium*) obtained on silver nanorod substrates, finding that they had reproducibility and showed the potential of the SERS technique in distinguishing different species, pure cell samples and mixed cell samples, despite their great similarity [11]. Since many bacterial groups in foodborne pathogenic bacteria have similar Raman characteristics, there may be errors in identifying specific bacterial groups. Appropriate multivariate statistical analysis methods could be used to improve the identification effect, such as principal component analysis (PCA), hierarchical cluster analysis (HCA) and linear discriminant analysis (LDA). For instance, Mircescu et al. [12] successfully classified *Staphylococcus aureus* (*S*. *aureus*), *Enterococcus faecalis*, and *Pseudomonas aeruginosa* into Gram types by combining PCA and LDA methods, while Liu et al. [13] used the SERS spectra combined with PCA and HCA to identify four *Pseudomonas* species isolated from chicken breast meat demonstrating that their genetic relationships matched with those obtained by 16S rRNA sequence analysis, achieving a classification accuracy of 100% and a rapid identification of bacteria. However, these studies mainly focused on the identification of bacteria at the species level or lower levels while ignoring the classification of bacteria at higher levels such as Gram type level or genus level.

This study aims to use the SERS technique combined with chemometrics methods to achieve rapid, sensitive, accurate and simple detection of the foodborne pathogenic bacteria *S*. *typhimurium*, *E*. *coli* O157:H7, *S*. *aureus*, and *Listeria monocytogenes* (*L*. *monocytogenes*), and two kinds of non-pathogenic bacteria, *Listeria innocua* (*L*. *innocua*), and *Listeria welshimeri* (*L*. *welshimeri*) at different classification levels.

## 2. Materials and Methods

### 2.1. Test Strains and Chemicals

Test strains of *S*. *typhimurium* ATCC 14028, *E*. *coli* O157:H7, S2, *S*. *aureus* ATCC 25923, *L*. *monocytogenes* ATCC 19115, *L*. *innocua* ATCC 33090, and *L*. *welshimeri* CICC 21672 were isolated from beef samples and provided by the Laboratory of Beef Processing and Quality Control, College of Food Science and Engineering, Shandong Agriculture University. Among them, *S*. *aureus* and *L*. *monocytogenes* are Gram-positive pathogenic bacteria, *S*. *typhimurium* and *E*. *coli* are Gram-negative pathogenic bacteria, and *L*. *innocua* and *L*. *welshimeri* are non-pathogenic bacteria. Silver nitrate (99%) and sodium citrate (99%) were purchased from Sinopharm Chemical Reagent Co. Ltd. (Shanghai, China). All other chemicals were of analytical grade and used without further purification.

### 2.2. Culture of Single Bacteria

The frozen *S*. *typhimurium*, *E*. *coli* O157:H7, *S*. *aureus*, *L*. *monocytogenes*, *L*. *innocua*, and *L*. *welshimeri* were inoculated into fresh BHI medium and shaken for 18 h (200 r/min, 37 °C) to activate them. The bacterial suspensions were centrifuged at 6000× rpm 4 °C for 10 min after twice activations. The medium was removed, and the bacteria were washed three times with sterile saline. The bacteria were resuspended in sterile saline to obtain bacterial suspensions with a bacterial count of about 10^8^ colony forming units per millilitre.

### 2.3. Preparation of AgNPs

AgNPs were prepared following the Lee-Meisel method [14] of sodium citrate reduction. To this end, silver nitrate (0.018 g) was dissolved in ultrapure water (100 mL), and the solution was brought to a boil on an electric stove. Then, 2 mL of sodium citrate of different concentrations was added, and the mixture was heated under reflux on a constant magnetic stirrer at a constant temperature. The solution was kept boiling and stirred for 60 min until the silver nitrate and sodium citrate in the solution were completely decomposed to mitigate the background interference in Raman spectroscopy, and the silver nanosol containing AgNPs was obtained. The prepared silver nanosol was naturally cooled to room temperature, and after centrifugal washing with ultrapure water three times, the silver nanosol was resuspended with ultrapure water of equal volume and stored in the dark at 4 °C.

The AgNPs were centrifuged at 7000 rpm for 5 min. The supernatant was then discarded. The precipitate was dissolved in pure water until it became clear and transparent. The dissolved precipitate was dropped onto a 5 × 5 mm silicon wafer. It was left to dry naturally to form a film. After drying, the sample was prepared for gold sputtering using a vacuum sputter coater. It was placed on the sample stage, approximately 10 to 15 cm from the evaporation source. The sample was rotated during the sputtering process. Uniformly apply a thin layer of gold through sputtering at 10 kV for 60 s, and once the process is finished, examine the sample using the GeminiSEM300 scanning electron microscope (SEM) manufactured by Carl Zeiss AG in Oberkochen, Germany. Due to the precipitation of AgNPs during storage, the prepared silver nanosol was fully mixed before use.

### 2.4. Pre-Treatment and Condition Optimisation of SERS Detection Samples

#### 2.4.1. Optimisation of the Size of AgNPs

Given that the size of the obtained AgNPs could be changed by varying the concentration of sodium citrate solution added to the silver nitrate solution in the preparation process of silver nanocolloid [15], 2 mL of sodium citrate solution with concentrations of 3%, 2%, and 1% were separately added to the silver nitrate solution to prepare silver nanocolloids with different sizes of AgNPs. The AgNPs-bacteria complexes with different sizes were then dropped onto quartz cuvettes and scanned by Raman spectroscopy.

#### 2.4.2. Optimisation of the Volume Ratio of AgNPs and Bacterial Suspension

The prepared silver nanocolloid and bacterial suspension, which had a bacterial count of about 8 log CFU/mL, were mixed at volume ratios of 5:1 (5 mL:1 mL), 2:1 (4 mL:2 mL), 1:1 (3 mL:3 mL), and 0.5:1 (4 mL:2 mL), respectively. The mixture was shaken and incubated at room temperature for 30 min in order to improve the binding of bacteria with AgNPs. The bacteria-AgNPs complex was then scanned by Raman spectroscopy.

### 2.5. Acquisition of Raman Spectra

Raman spectra of the bacteria-AgNPs complexes were collected using a Raman Pro portable Raman spectrometer (B&WTek, LLC, Plainsboro Township, NJ, USA) with an excitation wavelength of 532 nm, laser power of 14 mW, using a 20× objective lens, integration time of 1 s, integration number of 10, and Raman shift acquisition range between 400 and 1800 cm^−1^. During the SERS spectral scanning of six strains of bacteria, 20 parallel samples were prepared for each strain, and ten Raman spectra were scanned for each sample, with a total of three experiments conducted.

### 2.6. Preprocessing of Raman Spectra

Before building the chemometric model, the data were preprocessed according to the method of Liu et al. [16]. The preprocessing methods applied to Raman spectra signals were SNV (standard normal variate) and first-order derivative. Before performing these two preprocessing methods, the spectra were smoothed using the Lowess method to improve the smoothness of the spectra and reduce the interference of noise.

### 2.7. Multivariate Statistical Analysis

To analyse the spectral data and classify the bacteria at different levels, unsupervised pattern recognition methods of PCA and HCA were used. PCA is a technique that transforms multidimensional spectral data that may be correlated into several linearly uncorrelated variables, which are the principal components (PCs), which retain the information contained in the original spectra [13,17,18]. Consequently, PCA can reduce the Raman spectral data from hundreds or thousands of dimensions to three-dimensional space and visualise the distribution and clustering of the data using a three-dimensional principal component score plot [19]. HCA is a clustering method based on the similarity between data elements, which can classify the samples according to different distance measures and form a dendrogram to show the relationship between the samples [20]. In this study, we chose the Mahalanobis distance as the distance measure for HCA and obtained a cluster analysis dendrogram to perform analysis at different levels. Besides PCA and HCA, LDA was also utilised as another multivariate statistical analysis method to determine the type of samples. The purpose of LDA is to establish a discriminant function based on known classifications and predict the category of newly observed objects. Data analysis was completed using Matlab R2016a for analysis and processing, while the particle size distribution diagram was drawn using the Nano Measurer 1.2.5 software.

## 3. Results and Discussion

### 3.1. SERS Detection Optimisation with AgNPs Substrates

#### 3.1.1. Analysis of AgNPs Size Optimization on SERS

**Figure 1:**
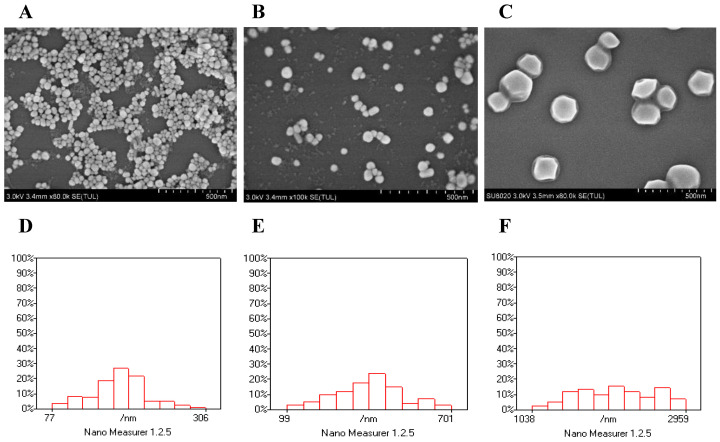
SEM images (**A**–**C**) and particle size distribution (**D**–**F**) of AgNPs prepared with different concentrations of sodium citrate. (**A**,**D**): 3% sodium citrate; (**B**,**E**): 2% sodium citrate; (**C**,**F**): 1% sodium citrate. In (**D**–**F**), the x-axis shows AgNP size in nm, and the y-axis shows the relative percentage of AgNPs.

In the preparation process of silver nanocolloids, the size of AgNPs could be changed by changing the concentration of sodium citrate added. In this experiment, 3%, 2%, and 1% sodium citrate were used to reduce silver nitrate and obtain silver nanocolloid. Figure 1A–C shows the SEM images of AgNPs with three different sizes. It can be seen from the SEM images that the size of AgNPs changed with the change in the sodium citrate concentration. The AgNPs with 3% sodium citrate had the smallest size, followed by those with 2% sodium citrate, and those with 1% had the largest size. As shown in Figure 1D, the size of AgNPs with 3% sodium citrate was mostly distributed in the range between 140 and 200 nm, and the average size was calculated to be 170 ± 7 nm. As shown in Figure 1E, the size of AgNPs with 2% sodium citrate was mostly distributed in the range between 250 and 500 nm, and the average size was calculated to be 300 nm (±7 nm). As shown in Figure 1F, the size of AgNPs with 1% sodium citrate was mostly distributed in the range between 1400 and 2200 nm, and the average size was calculated to be 1600 nm (±7 nm).

The experimental results showed that adding different concentrations of sodium citrate could change the size of AgNPs. Subsequently, the SERS spectra of *S*. *typhimurium* combined with AgNPs with three different sizes were scanned, and the most suitable AgNP size for the SERS detection of pathogenic bacteria was selected according to their enhancement degree of Raman signals.

**Figure 2:**
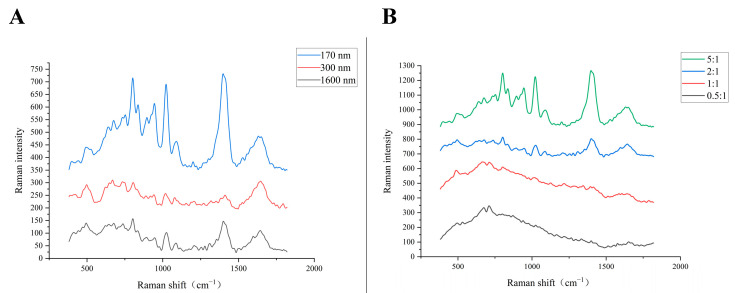
SERS spectra of AgNPs with different particle sizes (**A**) and different volume ratios of nanosilver sol and bacterial solution (**B**) as enhanced substrates. (**A**): blue line: average particle diameter of 170 nm; red line: average particle diameter of 300 nm; black line: average particle diameter of 1600 nm. (**B**): green line: 5:1; blue line: 2:1; red line: 1:1; black line: 0.5:1.

The SERS spectra with different sizes of AgNPs as enhancement substrates are shown in Figure 2A. At 493 cm^−1^, 799 cm^−1^, 1026 cm^−1^ and 1395 cm^−1^, there were peaks with different signal intensities in the three Raman spectra. It can be seen that the SERS spectrum with AgNPs of 170 nm as enhancement substrate had significantly stronger Raman signals at the above four Raman shifts than the other two SERS spectra with different sizes of substrates. Therefore, the succeeding experiments used AgNPs of 170 nm, which were prepared by adding 3% sodium citrate as a SERS enhancement substrate for further detection, analysis, and identification.

#### 3.1.2. Analysis of Volume Ratio Optimization on SERS

According to the results in Section 3.1.1, the AgNPs of 170 nm size were used as the SERS enhancement substrate for the optimisation of the volume ratio of silver nanocolloid and bacterial suspension. Comparing the enhancement effects of different volume ratios of silver nanocolloid and bacterial suspension, which were 5:1, 2:1, 1:1, and 0.5:1 (Figure 2B) with the increase of the volume ratio of silver nanocolloid and bacterial suspension, the intensities of the Raman shift at 493 cm^−1^, 799 cm^−1^, 1026 cm^−1^ and 1395 cm^−1^ also increased. Consequently, for further experiments, we used a volume ratio of silver nanocolloid and a bacterial suspension of 5:1 for detection, analysis and identification.

In this study, the influence of various particle sizes of AgNPs on their effectiveness as SERS enhancement substrates for bacteria was explored, revealing that AgNPs with a size of 170 nm significantly amplified the Raman signal intensity. This outcome aligns with the principle established by Wei et al. [21], which posits that specific particle sizes may optimise the enhancement effect, potentially due to the interplay between the increased specific surface area and the local electric field intensity that such particle sizes afford. The study by Wei et al. [21] demonstrated how the size of nanoparticles can be tailored to enhance the SERS signals, a concept mirrored in our findings with the 170 nm AgNPs. In addition, the optimal SERS spectral signal could be obtained when the volume ratio of silver nanocolloid to bacterial solution was 5:1. This agrees with Guicheteau et al. [22], who suggested that more silver nanoparticles binding to bacteria could effectively enhance Raman scattering.

### 3.2. Establishment of Bacterial Classification Model

#### 3.2.1. Bacterial Gram Type Classification

**Figure 3:**
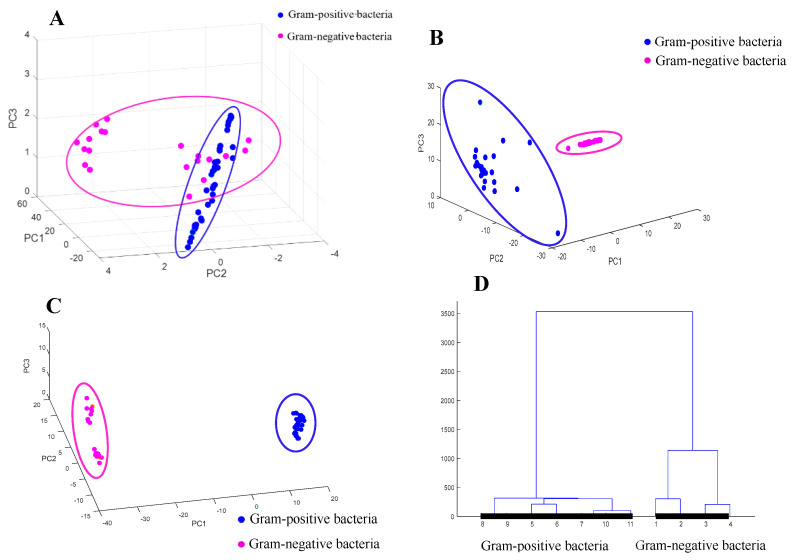
Raman spectra of bacteria at the Gram staining level (**A**), PCA classification results of the corresponding Raman spectra after preprocessing (**B**,**C**), and HCA plot (**D**). (**A**) Original Raman spectra of bacteria; (**B**) Raman spectra after first-order derivative preprocessing; (**C**) Raman spectra after SNV preprocessing; (**D**) HCA plot of Gram-positive and Gram-negative bacteria.

Figure 3A shows the classification results of the original spectra of the six strains of bacteria at the Gram type level using PCA. It can be seen that there was a certain degree of classification trend between Gram-positive and Gram-negative bacteria, yet samples of different Gram types were overlapping, consequently the Gram-positive and Gram-negative bacteria were not adequately separated.

The classification results of the original Raman spectra of the six bacteria at the Gram type level using PCA after first-order derivative and SNV preprocessing are depicted in Figure 3B,C. These figures illustrate the effective separation of the two Gram types achieved by the PCA following the application of the two preprocessing methods. First-order derivative preprocessing is a commonly used spectral derivative technique that enhances spectral features and reduces the impact of baseline drift by calculating the derivative of spectral data [16]. It is capable of eliminating constant baseline offsets, and its primary purpose is to remove additive and multiplicative effects in the spectra, thereby making the characteristics of the analyte more pronounced. However, the clustering effect of the Raman spectral data after first-order derivative preprocessing was inferior compared to SNV preprocessing, with many scattered points exhibiting large distances. In contrast, the clustering effect of the spectral data after SNV preprocessing was clearer than that of the first-order derivative preprocessing. SNV is a preprocessing technique aimed at reducing the physical variability caused by scatter among samples [23]. In SNV, each spectrum is centred and then scaled according to its corresponding standard deviation, resulting in each sample having a unit standard deviation. The main function of SNV is to eliminate baseline drift while reducing the multiplicative effects of scatter, making the spectral data more consistent. Consequently, the enhanced pattern recognition effects were clearly demonstrated through SNV preprocessing, and the PCA proved more effective in distinguishing between the two Gram types of bacteria. The better spectral aggregation following SNV preprocessing shows that the results were improved.

Four Gram-negative and seven Gram-positive bacteria were randomly selected for HCA analysis, and a cluster analysis dendrogram was obtained. As shown in Figure 3D, the spectral samples of bacteria with the same Gram type were clustered together. Among them, No. 1 to No. 4 were Gram-positive bacteria, and No. 5 to No. 11 were Gram-negative bacteria. Therefore, it could be concluded that the results of the PCA and HCA were consistent, and they both achieved the classification of different strains at the Gram type level, laying a foundation for the following classification and identification at the genus and species levels.

#### 3.2.2. Bacterial Genus Level Classification

**Figure 4:**
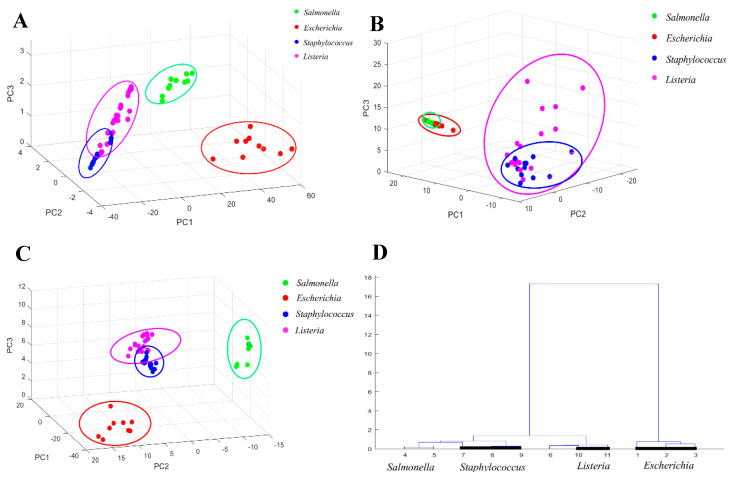
PCA classification and HCA cluster analysis results of Raman spectra at the bacterial genus level (**A**–**D**). (**A**) PCA classification results of raw Raman spectra; PCA classification results of Raman spectra after (**B**) first-order derivative and (**C**) SNV preprocessing; (**D**) dendrogram of HCA clustering analysis for four genera of bacteria.

The *S*. *typhimurium*, *E*. *coli* O157:H7, *S*. *aureus*, *L*. *monocytogenes*, *L*. *innocua*, and *L*. *welshimeri* used in this study, belong to four different genera, *Salmonella*, *Escherichia*, *Staphylococcus* and *Listeria*. Figure 4A shows the classification results of the original spectra of the six strains of bacteria at the genus level using the PCA; from this, the samples of the same genus were clustered together. *Escherichia* and *Salmonella* were adequately classified and separated from *Staphylococcus* and *Listeria*. Although there was a classification trend between *Staphylococcus* and *Listeria*, there was still some overlap. The results highlighted that the direct PCA of the original spectra could not separate the four genera, and further processing was needed to explore the separation and classification results after different preprocessing.

The classification results of PCA after first-order derivative preprocessing and SNV preprocessing for the four genera are given in Figure 4B and 4C, respectively. As illustrated by Figure 4B, the samples after first-order derivative preprocessing had a large degree of dispersion after PCA, and the separation effect of the four genera was poor. *Escherichia* and *Salmonella* had a separation trend, but there was still some overlap. While these two genera were separated from the other two genera, *Staphylococcus* and *Listeria* had a large degree of overlap and could not be separated at all. Figure 4C shows the classification results of PCA after SNV preprocessing of the samples. It is evident from the figure that the samples of the four genera were completely separated, and each genus had an efficient clustering effect, as while a few samples of *Staphylococcus* and *Listeria* overlapped, they were generally separated. Thus, it can be concluded the PCA results after SNV preprocessing were more accurate than those after first-order derivative preprocessing. The Raman spectra of different genera of foodborne pathogenic bacteria could be adequately classified, and this classification could be applied to the subsequent analysis at the species level.

As illustrated in Figure 4D, a total of 11 spectra from four genera were randomly selected for cluster analysis. Among them, *Escherichia* had three spectra, numbered 1 to 3; *Salmonella* had three spectra, numbered 4 to 7; *Staphylococcus* had three spectra, numbered 7 to 9; *Listeria* had two spectra, numbered 10 to 11. As highlighted in Figure 4D, the samples of *Escherichia*, *Staphylococcus*, and *Listeria* were clustered together, but *Salmonella* was scattered. Only No. 4 and 5 samples were clustered together, while No. 6, which also belonged to *Salmonella*, was wrongly clustered with two *Listeria* samples. The classification results showed that although there were some misclassifications, the overall clustering effect was robust. The cluster analysis method could generally classify the four genera.

From the above analysis results, it could be concluded that the results of PCA and HCA were mainly consistent, both achieving the classification of six bacteria at four genus levels, laying a foundation for the following classification and identification at the species level.

#### 3.2.3. Bacterial Species Level Classification

**Figure 5:**
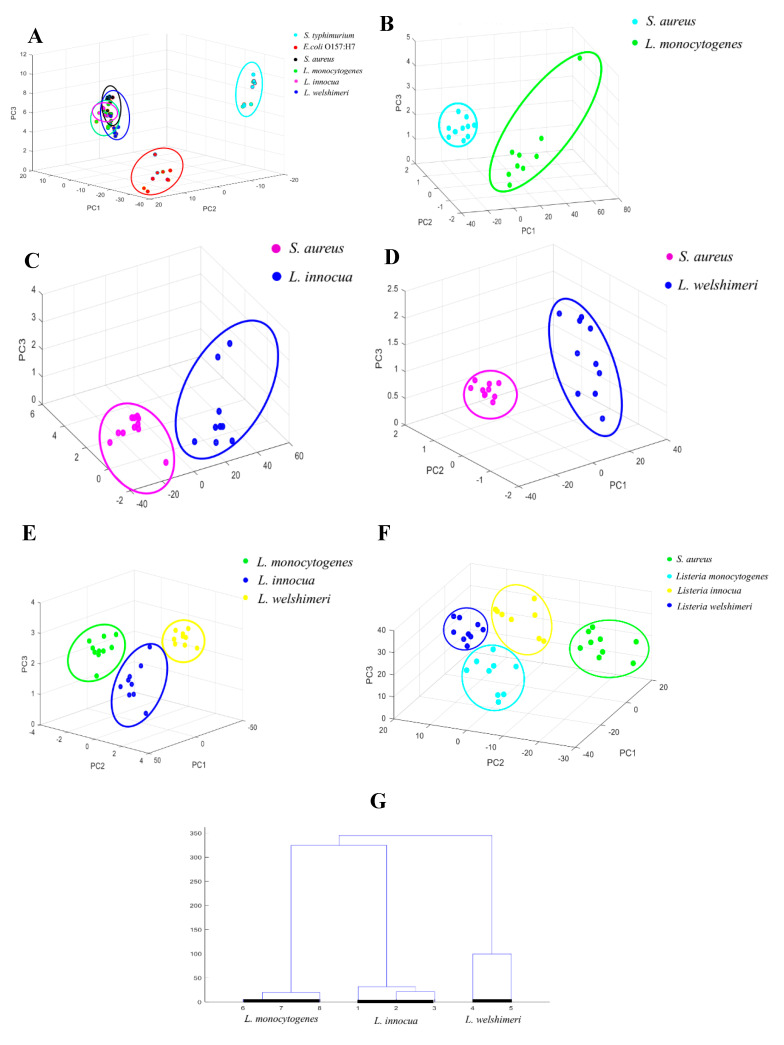
PCA classification and HCA cluster analysis results of Raman spectra at the bacterial species level (**A**–**G**). (**A**) PCA classification results of bacterial raw Raman spectra at the species level; PCA results of (**B**) *S. aureus* and *L. monocytogenes*; (**C**) *S. aureus* and *L. innocua*; (**D**) *S. aureus* and *L. welshimeri*; (**E**) *L. monocytogenes*, *L. innocua* and *L. welshimeri*; (**F**) PCA classification results of SNV preprocessed Raman spectra; (**G**) dendrogram obtained using HCA of *Listeria* species.

Figure 5A shows the classification results of the original Raman spectra of *S*. *typhimurium*, *E*. *coli* O157:H7, *S*. *aureus*, *L*. *monocytogenes*, *L*. *innocua*, and *L*. *welshimeri* using PCA. The comparison results are shown in Figure 5B–E, which respectively represent *S*. *aureus* and *L*. *monocytogenes*, *S*. *aureus* and *L*. *innocua*, *S*. *aureus* and *L*. *welshimeri* and three strains of *Listeria* including *L*. *monocytogenes*, *L*. *innocua*, and *L*. *welshimeri*.

From Figure 5A, it is evident the Raman spectra samples belonging to *S*. *Typhimurium* and *E*. *coli* O157:H7 were relatively independently clustered and separated from the other five bacterial species. However, the sample points of the Raman spectra of *S*. *aureus*, *L*. *monocytogenes*, *L*. *innocua*, and *L. welshimeri* overlapped with four bacterial species that were not separated at all. Therefore, *S*. *aureus*, *L*. *monocytogenes*, *L*. *innocua*, and *L*. *welshimeri* were separately subjected to pairwise PCA to explore their bacterial species classification and the classification effect between pathogenic and non-pathogenic bacteria.

When the original Raman spectra of six strains of bacteria were simultaneously subjected to PCA, except for *S*. *typhimurium* and *E*. *coli* O157:H7, which could be separated, the bacterial species, including *S*. *aureus*, *L*. *monocytogenes*, *L*. *innocua*, and *L*. *welshimeri* had a high degree of overlap and could not be separated from each other. Therefore, the original spectra of these four bacterial species were separately compared by pairwise PCA. From Figure 5B–E, it is evident that when the bacterial species that could not be separated initially were put together in pairs for PCA comparison, they could be separated, and the clustering effect of the same bacterial species was improved without any overlap. Therefore, the separation effect was superior to that of the six bacterial species in the PCA. Consequently, different bacterial species could be classified by reducing the number of bacterial species to improve the classification effect of PCA. After performing pairwise PCA on the original Raman spectra of the above four bacterial species separately, an attempt was made to perform SNV preprocessing on the original spectra of these four bacterial species and then compare them by a PCA to observe the classification results. In the classification at the genus level, the classification effect after SNV preprocessing was significantly better than that after first-order derivative preprocessing. Therefore, SNV preprocessing was chosen to perform PCA on these four bacterial species at the species level.

Figure 5F shows a PCA result diagram of the Raman spectra of *S*. *aureus*, *L*. *monocytogenes*, *L*. *innocua*, and *L*. *welshimeri* after SNV preprocessing. From the figure, it is obvious that the SNV preprocessing method greatly improved the clustering degree of four bacterial species and through PCA, the four bacterial species could be completely separated. Moreover, each sample point of each bacterial species was independent without any overlap. Subsequently, the classification and identification of these four bacterial species, as well as pathogenic *Listeria* and non-pathogenic *Listeria*, could be achieved. Therefore, it was concluded that the SNV preprocessing method could improve the clustering degree of samples from the same bacterial species and facilitate a more accurate classification of different bacterial species.

After successfully separating different bacterial species by PCA, the classification situation of pathogenic *L*. *monocytogenes* and non-pathogenic *Listeria* bacteria, three *L*. *monocytogenes* bacteria were randomly selected. Three *L*. *innocua* bacteria and two *L*. *welshimeri bacteria* were selected for Raman spectral data analysis using HCA. As illustrated in Figure 5G, *Listeria* bacteria of the same species were all clustered together; among them, No. 1 to 3 were *L*. *innocua* bacteria; No. 4 to 5 were *L*. *welshimeri* bacteria; and No. 6 to 8 were *L*. *monocytogenes* bacteria. Consequently, it was concluded that the PCA and HCA results were consistent and could classify the Raman spectra of different foodborne pathogenic bacteria and separate pathogenic *L*. *monocytogenes* from the other two non-pathogenic *Listeria* bacteria.

This study revealed the differential characteristics of different foodborne pathogenic bacteria on Raman spectra, which provided a reference for further exploring the relationship between bacterial Raman spectra and their composition, structure, and function. For instance, according to Liu et al. [24], five spore-forming bacteria (*Clostridium perfringens*, *Bacillus subtilis*, *Bacillus amyloliquefaciens*, *Bacillus licheniformis*, and *Paenibacillus pabuli*) isolated from three spice powders showed significant differences in their Raman spectra, reflecting their biochemical differences in nucleic acids, proteins, and saccharides. The Raman spectra of these spore-forming bacteria exhibited different intensity changes at wavelengths such as 552–559 cm^−1^, 1001–1006 cm^−1^, 1092–1097 cm^−1^, 1663–1674 cm^−1^, which corresponded to the characteristic peaks of carbohydrates, proteins, nucleic acids, and unsaturated lipids, respectively. These spectral differences could be used to distinguish different phylogenetic groups and provide technical support for the rapid and accurate detection of foodborne bacteria. At the genus level, the SERS spectra of *E*. *coli* and *S*. *typhimurium* could be used to distinguish these two species by applying PCA–HCA, as they showed obvious differences in the regions between 600 and 800 cm^−1^, 1000 and 1200 cm^−1^, and 1400 and 1600 cm^−1^, which corresponded to the vibrations of nucleic acids, amino acids and lipids, and a combination of nucleic acids and amino acids, respectively [25]. Similarly, *L*. *monocytogenes* demonstrated unique peaks at 627 cm^−1^ (phenylalanine skeletal vibration), 732 cm^−1^ (glycosidic ring/adenine/CH_2_ rocking), and 1097 cm^−1^ (C-C skeletal and COC stretching from the glycosidic link), offering a clear spectral signature for this bacterium [26]. At the species level, even bacteria of the same genus, due to the existence of minor genetic variations or phenotypic changes, would cause subtle differences in Raman spectra [27,28]. Saba Bashir et al. [29] used the SERS spectroscopy technique, combined with chemometrics methods such as PCA, HCA and PLS-DA, to qualitatively and quantitatively distinguish and diagnose different strains of *E*. *coli*. Sundaram et al. [30] used SERS spectra to detect and differentiate three different serotypes of *Salmonella* and found that using biopolymer coated silver nanoparticles as SERS substrates could improve their detection accuracy. Their study showed that SERS spectra could effectively discriminate between three serotypes of *Salmonella* cells with up to 98% classification under the PCA model. Therefore, by analysing the Raman spectra of different foodborne pathogenic bacteria in depth, the intrinsic relationship between these bacteria and their biological characteristics can be revealed, providing valuable information for understanding various features of bacteria, including physiological metabolism, adaptability, drug resistance, and more.

### 3.3. LDA

The supervised pattern recognition method, LDA, was employed to analyze 540 randomly selected spectral data. This dataset included 360 spectra for the training set and 180 spectra for the test set, which were used to verify the accuracy and stability of the PCA and HCA results. These findings are detailed in Table 1.

In the training set, among the 60 spectra of *E*. *coli* O157:H7, only three spectra were misclassified as *S*. *typhimurium*, with a spectral recognition accuracy of 95%. Among the 60 spectra of *L*. *monocytogenes*, four were misclassified as *S*. *aureus*, four as *L*. *welshimeri*, and five as *L*. *innocua*, with a spectral recognition accuracy of 89%. All 240 spectra of *S*. *typhimurium*, *S*. *aureus*, *L*. *innocua*, and *L*. *welshimeri* were correctly assigned, with a spectral recognition accuracy of 100%. In the prediction set, *S*. *typhimurium*, *E*. *coli* O157:H7 and *S*. *aureus* were correctly discriminated. Among the 30 spectra of *L*. *monocytogenes*, one was misclassified as *S*. *aureus* and three as *L*. *welshimeri*, resulting in a discrimination accuracy of 87%. Among the 30 spectra of *L*. *innocua*, four were misclassified as other bacterial species, and the remaining 26 spectra were correctly discriminated; consequently, the discrimination accuracy was 87%. Among the 30 spectra of *L*. *welshimeri*, three were misclassified as *L*. *innocua* and the others were correctly discriminated, with a discrimination accuracy of 90%. Therefore, the classification accuracy of the training set was 94%. Overall, LDA achieved a spectral recognition accuracy of 95.65% for the whole data set.

When the optimised SERS detection method was used with PCA and HCA, it was possible to achieve the classification and identification of six foodborne pathogenic bacteria at three levels: Gram type, genus and species. Using LDA to validate the classification results demonstrated the success of the method, as an average accuracy of 95.65% was achieved. This result indicates that the SERS technology combined with chemometrics methods can effectively distinguish different types, genera and species of bacteria and has high sensitivity and specificity. To further evaluate the performance of the method under investigation, a comparison was made with the SERS-based aptasensor method developed by Duan et al. [31], which also targeted *S*. *typhimurium* as one of the pathogenic bacteria. Our method can identify multiple types of bacteria, while the aptasensor method is specific to one type of bacteria. Therefore, our method has a broader application range and can be used for rapid screening of various foodborne bacteria. Similarly, Mungroo et al. [32] and Srividya et al. [33] suggested using different chemometrics methods to analyse SERS spectral data to achieve consistent classification effects. Moreover, our method improved the resolution of SERS technology in bacterial detection by adding classification at the genus level, which was not achieved by Chu et al. [11], who only showed the classification results at the Gram type and species level based on the SERS spectra from whole cell bacteria, although they suggested that the genus level classification might be possible. This improvement in resolution is in agreement with studies such as Meisel et al. [34], who also used the SERS technology to quickly identify meat-related bacteria and established a three-level classification model. For the detection of pathogenic bacteria species on certain meat products, in addition to the low detection limit of these bacteria, the ability to distinguish different bacterial genera is also essential. Breuch et al. [35] found that using the SERS technique could achieve the rapid, non-destructive identification of seven important meat-related microorganisms and quickly detect spoilage bacteria and foodborne pathogens.

Although this study demonstrated the potential for using AgNPs and SERS to detect microbial species, our findings are limited by the small number of bacterial species used in this study. While the six foodborne pathogenic bacteria used were all common bacteria, findings may not be transferrable given more types and more complex bacteria are expected in industrial applications. In order to improve the application scope and accuracy of the SERS technique in bacterial detection, it will be necessary to scan and analyse more types and more sources of bacteria by SERS spectroscopy to establish a complete and more comprehensive bacterial spectral database and classification model [36].

## 4. Conclusions

This study leveraged SERS technology in conjunction with chemometric approaches to achieve rapid and precise detection and classification of six foodborne pathogens in beef samples. The use of AgNPs as SERS substrates notably amplified the Raman signal intensity of bacteria. By modulating the concentration of sodium citrate, AgNPs of varying sizes were synthesised, with those prepared using 3% sodium citrate (average size of 170 nm) demonstrating the optimal enhancement of Raman signals in SERS detection. PCA and HCA were employed to construct classification models across three levels: Gram type, genus, and species. These models were subsequently validated using LDA on a dataset of 540 spectra, yielding an average accuracy of 95.65%. Our findings suggest that the integration of SERS with chemometric methods enhances both the sensitivity and specificity of bacterial detection while streamlining the process, offering a potent tool for food safety surveillance. Moreover, the outcomes of this study furnish a scientific foundation for the broader application of SERS in food microbiological detection and classification, holding significant implications for the advancement of rapid microbial detection methodologies within the food industry.

## Figures and Tables

**Table 1:** LDA analysis of Raman spectral data of six strains of bacteria.

Bacteria	Training Set (*n* = 360)							Test Set (*n* = 180)						
	*S.ty*	*E.co*	*S.au*	*LM*	*LIN*	*LWE*	Accuracy (%)	*S.ty*	*E.co*	*S.au*	*LM*	*LIN*	*LWE*	Accuracy (%)
*S.ty*	60	0	0	0	0	0	100	30	0	0	0	0	0	100
*E.co*	3	57	0	0	0	0	95	0	30	0	0	0	0	100
*S.au*	0	0	60	0	0	0	100	0	0	30	0	0	0	100
*LM*	0	0	4	49	5	4	89	0	0	1	26	3	0	87
*LIN*	0	0	0	0	60	0	100	0	0	1	2	26	1	87
*LWE*	0	0	0	0	0	60	100	0	0	0	0	3	27	90
Overall accuracy							97.3							94
Average accuracy			95.65											

Note: *S.ty*: *S. typhimurium*; *E.co*: *E.coli* O157:H7; *S.au*: *S. aureus*; *LM*: *L. monocytogenes*; *LIN*: *L. innocua*; *LWE*: *L. welshimeri*.

## Data Availability

The original contributions presented in this study are included in the article. Further inquiries can be directed to the corresponding author.
